# Hospital-derived antibody profiles of malaria patients in Southwest India

**DOI:** 10.1186/s12936-019-2771-5

**Published:** 2019-04-17

**Authors:** Apoorva Venkatesh, Aarti Jain, Huw Davies, Ligia Periera, Jennifer N. Maki, Edwin Gomes, Philip L. Felgner, Sanjeeva Srivastava, Swati Patankar, Pradipsinh K. Rathod

**Affiliations:** 1 Department of Biosciences and Bioengineering, Indian Institute of Technology Bombay, Mumbai, India; 2 Department of Medicine, Goa Medical College and Hospital, Bambolim, Goa 403202 India; 3 Department of Chemistry and Department of Global Health, University of Washington, Seattle, WA 98195 USA; 4 Vaccine R&D Center, University of California, Irvine, CA 92697 USA

**Keywords:** Seroreactive antigens, *Plasmodium vivax*, *Plasmodium falciparum*, Malaria exposure, Protective immunity, Protein arrays

## Abstract

**Background:**

Naturally acquired immunity to malaria across the globe varies in intensity and protective powers. Many of the studies on immunity are from hyperendemic regions of Africa. In Asia, particularly in India, there are unique opportunities for exploring and understanding malaria immunity relative to host age, co-occurrence of *Plasmodium falciparum* and *Plasmodium vivax* infections, varying travel history, and varying disease severity. Variation in immunity in hospital settings is particularly understudied.

**Methods:**

A US NIH ICEMR (South Asia) team examined the level of immunity in an Indian malaria patient population visiting or admitted to Goa Medical College and Hospital in Goa, India. Sera from 200 patients of different ages, in different seasons, infected with *P. falciparum* or *P. vivax* or both species, and with different clinical severity were applied to an established protein array system with over 1000 *P. falciparum* and *P. vivax* antigens. Differential binding of patient IgG to different antigens was measured.

**Results:**

Even though Goa itself has much more *P. vivax* than *P. falciparum*, IgG reactivity towards *P. falciparum* antigens was very strong and comparable to that seen in regions of the world with high *P. falciparum* endemicity. Of 248 seropositive *P. falciparum* antigens, the strongest were VAR, MSP10, HSP70, PTP5, AP2, AMA1, and SYN6. In *P. vivax* patients, ETRAMPs, MSPs, and ApiAP2, sexual stage antigen s16, RON3 were the strongest IgG binders. Both *P. falciparum* and *P. vivax* patients also revealed strong binding to new antigens with unknown functions. Seropositives showed antigens unique to the young (HSP40, ACS6, GCVH) or to non-severe malaria (MSP3.8 and PHIST).

**Conclusion:**

Seroreactivity at a major hospital in Southwest India reveals antibody responses to *P. falciparum* and *P. vivax* in a low malaria transmission region with much migration. In addition to markers of transmission, the data points to specific leads for possible protective immunity against severe disease. Several, but not all, key antigens overlap with work from different settings around the globe and from other parts of India. Together, these studies confidently help define antigens with the greatest potential chance of universal application for surveillance and possibly for disease protection, in many different parts of India and the world.

**Electronic supplementary material:**

The online version of this article (10.1186/s12936-019-2771-5) contains supplementary material, which is available to authorized users.

## Background

Nearly half of the world’s population lives in malaria endemic zones. *Plasmodium falciparum* dominates in sub-Saharan Africa and accounts for the very high prevalence rates associated with this continent. *Plasmodium vivax* is mostly prevalent in the American continent and Asia [1]. Prevalence rates can mask the absolute amount of the disease. For instance, India has low endemicity due to the large population size but, as a country, India has the second highest number of malaria deaths in the world [1].

Within India, individuals are exposed to varying levels of endemicity and many develop partial protection against infection [2–4]. This naturally acquired immunity (NAI) is expected to vary with age, host genetic makeup, parasite species, and level of endemicity. In high transmission settings, individuals almost always have some levels of parasites in their blood. These asymptomatic carriers develop partial protection from severe disease at an early age, but rarely develop sterile immunity. In areas of low transmission, most individuals exhibit moderate to high-grade parasitaemia and develop clinical symptoms. Children are at a greater risk and are susceptible to severe disease until the age of about 2–3 years. Clinical immunity, although far from optimal, gradually develops with age, but lasts only as long as individuals are continuously exposed to and repeatedly infected with malaria parasites [5].

NAI is mediated by acquired immune mechanisms, primarily directed against blood stages of malaria parasites. Such immunity involves circulating immunoglobulins, antibody-producing plasma cells, and memory B cells [6–9]. Knowledge of dynamics underlying NAI is vital in identifying the most effective infection monitoring strategy for specific epidemiological settings. NAI can also be used to study the impact of control strategies on exposure and transmission. Antibodies exhibit several advantages over entomological and parasitological methods to estimate malaria exposure, prevalence, and transmission. In particular, antibodies provide evidence of exposure history since they persist in the body for some time. In an important technical advance, high-throughput protein arrays have been designed to study immunity against both *P. falciparum* and *P. vivax* in a cost-effective manner [10, 11]. A large collaborative effort was promoted on a global scale through the US NIH International Centres of Excellence for Malaria Research (ICEMRs). The efforts were expected to identify biosignatures of immunity, including the most informative antibody responses across a large number of malaria endemic sites. Using this approach, several groups coordinated investigations of antibody reactivity to hundreds of parasite antigens [10–19]. The studies were first performed using samples from 14 regions of the world with different epidemiological settings, transmission intensities and parasite prevalence. The trans-ICEMR survey used a standardized platform to measure antibody responses which was also used by other groups to study exposure and acquired immunity [15, 17, 18]. Using the standardized genome-scale protein arrays, the list of potential antigens that inform on malaria prevelance in global communities, prior exposure to malaria in individuals, and antibodies that affect infection, disease and transmission through mosquitoes has been greatly increased.

Immunity against malaria in India is less well understood compared to in Africa, SE Asia, or neighbouring islands. The effort above with the first protein arrays included a promising start to genome-scale probing of malaria immunity in India. An early comparative analysis of the seroreactivity profile from three sites, Raurkela, Nadiad, and Chennai in India indicated varied immune responses across the epidemiologically diverse endemic settings [19]. Raurkela and Nadiad are near rural sites with limited immigration from other parts of India. Chennai, which is in the deep south, has low level *P. vivax* and no local *P. falciparum*. Yet, importantly, asymptomatic carriers revealed higher reactivity to 19 *P. falciparum* antigens compared to symptomatic patients and they included PHISTc, MSP11, RH2b, RON2, SERA4, two VARs, RESA, ETRAMP5, MSP2, SEMP1, HSP70-x, GEXP18, GCN5, LSA3, ETRAMP14, MSP4, ring infected erythrocyte surface antigen and a conserved protein.

To assess the generality of the early findings from the studies in India and the world, and to understand and measure the level of exposure in low transmission areas, protein array-based immune surveillance was performed in a low epidemiological setting in Southwest India (Goa). While Goa is considered a non-malaria endemic region of India, it has significant local seasonal transmission of both *P. falciparum* and *P. vivax*. Most importantly, Goa Medical College and Hospital offers free health care to all and attracts a very wide spectrum of malaria patients. Many participants involved in the present study were immigrants from different parts of India, with different genetic backgrounds, and different histories of prior exposure to malaria [20]. These complex dynamics are typical of many large cities in central India and offer a chance to capture immune status of a wider population of infections in India. Collections at a hospital setting assured a broader set of disease presentations. In order to differentiate between recent and cumulative exposure, Immunoglobulin G (IgG) Ab responses in young children and adults were studied. Additionally, for the first time, a comparative analysis of IgG Ab levels in non-severe and severe malaria patients was performed to potentially discover new antigens of importance in the context of protection from severe disease.

In summary, the present study reveals, (i) seroreactivity profiles against both *P. falciparum* and *P. vivax* antigens of symptomatic malaria patients in Goa and (ii) the relationship of IgG antibody levels of this population in Goa to multiple factors that could contribute to differential immunity. These include their age, disease severity status, and season of sample collection. Such insights can potentially identify markers of exposure and disease severity that would be useful for malaria surveillance. The present results compare favourably with reactivity from non-urban areas of India and to the other ICEMR studies from around the world. This suggests that such antigens may have value as general sero-surveillance tools in very different epidemiological settings.

## Methods

### Ethics statement

The present study was a part of the US National Institutes of Health-sponsored Program Project entitled Malaria Evolution in South Asia-International Center of Excellence for Malaria Research (MESA-ICEMR). Samples were collected at Goa Medical College and Hospital (GMC), Bambolim, Goa, India. The sample collection process was approved by the Institutional Review Boards (IRB) of the Division of Microbiology and Infectious Diseases (DMID) at the US National Institute of Allergy and Infectious Diseases (NIAID), GMC, and the University of Washington (UW). Detailed ethics statements have been previously reported [20]. After sample collection and clearance from the Indian Institute of Technology, Bombay (IITB) Ethics Committee, the samples were transferred from Goa to IITB for microarray analysis. General patient information such as age, gender and time of sample collection, clinical information and results of laboratory tests for diagnosis were provided by the GMC and the UW teams to IITB for 200 patients. Other patient information remained classified to protect patient privacy.

### Study participants and sample classification

**Fig. 1:**
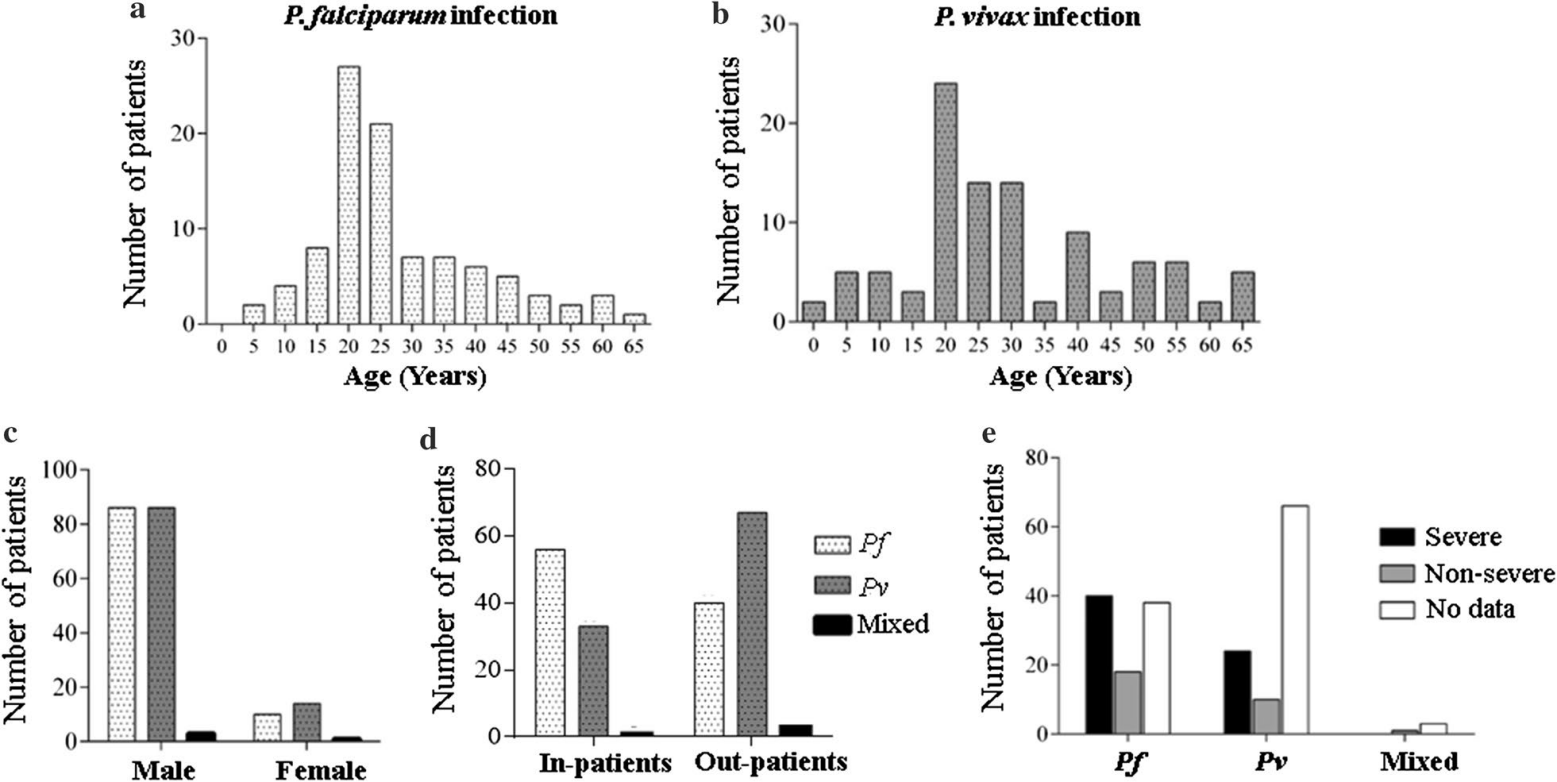
Study population (96 *P. falciparum*, 100 *P. vivax* and 4 mixed infections) classified based on age, gender, hospitalization status and severe symptoms. **a** Individual histograms for *P. falciparum* and **b** *P. vivax* patient groups show that a majority of the study participants were 20 to 25 years of age. Data for study group with mixed infection is not shown. **c** Bar graph of study participants indicate a male dominant population. **d** Plot represents hospitalization status for all three study groups. **e** Patients classified based on symptoms into severe and non-severe study groups (WHO classification for severe falciparum malaria)

Blood samples were collected from symptomatic malaria positive patients at GMC (Goa, India), year-round for 4 years (2012–2015). Written informed consent was obtained from all the volunteers during enrolment. A detailed description of the study site, enrolment and sample processing protocols has been published elsewhere [20]. Sera from 200 patients (96 *P. falciparum*, 100 *P. vivax,* and 4 mixed infections) were analysed by hybridization to protein microarrays. Parasite density (parasites/µL) was determined by microscopy for 199/200 individuals. Patients were further classified based on age (1–10 years, 11–22 years, 23–39 years and 40–65 years), hospitalization status, signs/symptoms of disease severity and time of sample collection (Fig. 1). Classification based on age was performed to segregate the population into young children, adolescents, young adults and older adults. WHO criteria for classification of severe *P. falciparum* malaria was used to define disease severity for both *P. falciparum* and *P. vivax* study groups due to the absence of specific case definitions for *P. vivax* malaria.

### Protein microarray probing with patient sera

Twenty microlitre (µL) aliquots of sera from 200 patients were transported to IITB in cryovials labelled with unique numerical codes, one for each patient. Antibody reactivity to *P. falciparum* and *P. vivax* proteins was studied using a protein microarray (*P. falciparum*/*P. vivax* 500) displaying 515 *P. vivax* and 500 *P. falciparum* proteins (Antigen Discovery, Inc., Irvine, CA). At ADI, the proteins were expressed as polypeptide fragments and printed from cell-free in vitro transcription translation (IVTT) reactions by the manufacturing team. A few large proteins such as dynein beta chains, rhoptry neck proteins, NOT family proteins, conserved *Plasmodium* proteins, reticulocyte binding proteins, erythrocyte membrane proteins (*Pf*EMP1) and a few transcription factors were printed as overlapping peptides or exon fragments. The antigens were down-selected from larger arrays (*Pf*4528 and *Pv*4441) based on the seroreactivity of different patient populations from other malaria endemic regions [16]. Antigens represent asexual parasite blood stages, preerythrocytic stages, and mixed stage proteins. Accession numbers and description of the polypeptides follow annotations published in PlasmoDB (http://www.plasmodb.org).

**Fig. 2:**
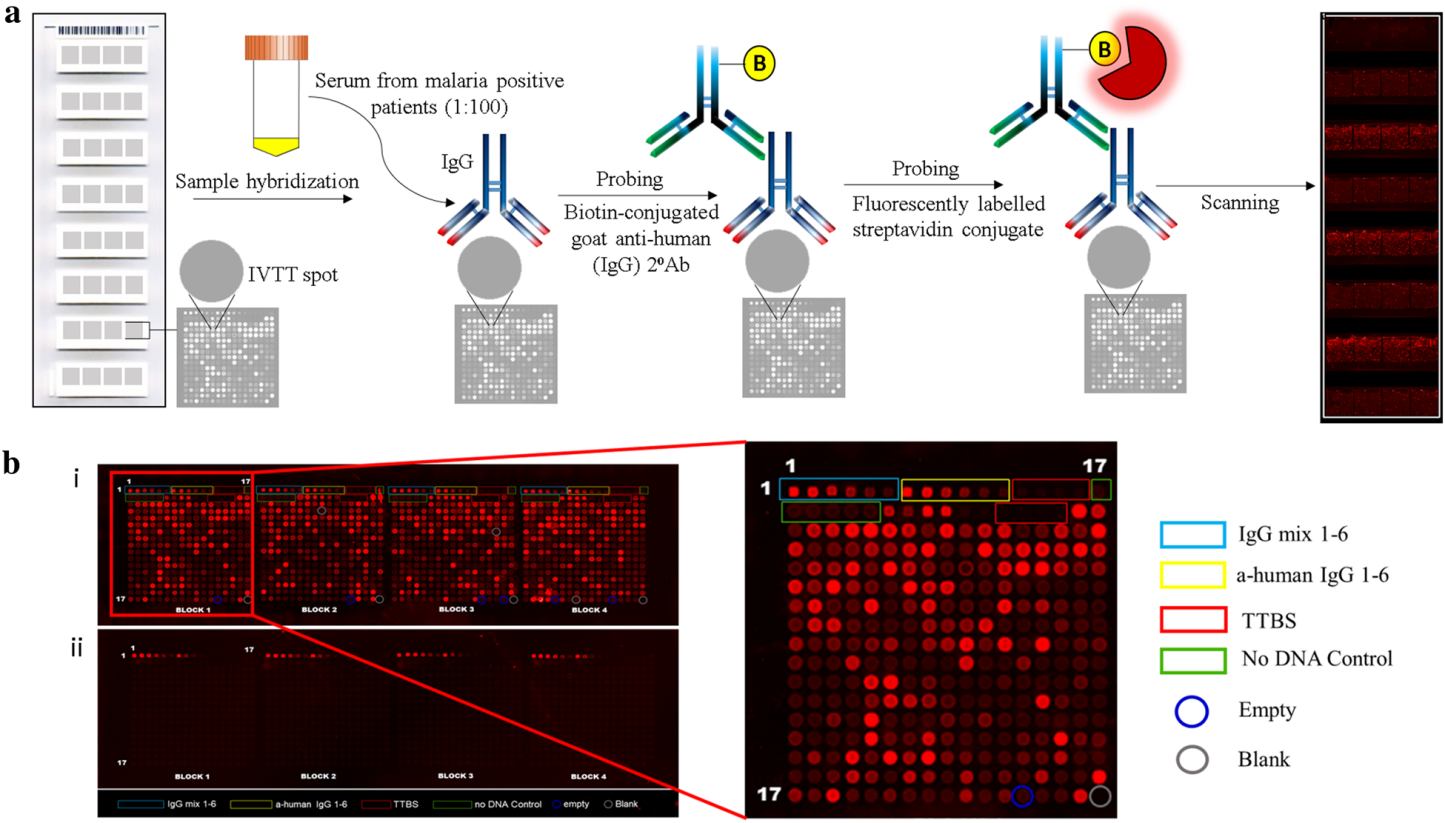
Protein microarray slide map. **a** Schematic representation of experiment design and workflow. **b** Scanned microarray pad probed with malaria positive patient sera (**i**) and no sera control probed with buffer alone (**ii**). A total of 500 *P. falciparum* and 515 *P. vivax* proteins were expressed as polypeptide fragments and printed as cell-free in vitro transcription translation (IVTT) reactions in four blocks. The pad also contains control spots; 24 IgG mix in six different concentrations (6 spots in each block), 24 anti-human IgG spots in six different concentrations, 32 TTBS (buffer) spots, 24 “No DNA-control” reaction spots lacking a DNA template in the plasmid vector, 7 blank and 6 empty spots arranged in four blocks (highlighted). 12 purified parasite proteins are printed in duplicates at two concentrations to serve as controls (printed between ‘No DNA’ control and TTBS spots)

Hybridizations were carried out at the Indian Institute of Technology Bombay (IITB). Each slide was composed of eight microarray pads. Multiple slides were used in each large experiment. Highly reactive sera from a single patient was used on one pad of every slide as a positive control. The rest of the microarray pads were used to probe sera from different patients. To track possible variations during array printing on individual pads on slides, batches of slides, or even assay performance during each experiment, select patient sera were probed across all runs and their signal intensities were compared for quality control and reproducibility. To account for non-specific reactivity of the IVTT expression system, each array pad contained 24 “No DNA-control” reaction spots, lacking a DNA template in the plasmid vector (Fig. 2). This ‘background signal’ was used for sample-specific normalization. Signals from immunoglobulin G (IgG) spots and anti-human IgG spots were used as standards to establish the correct PMT and laser power settings for each batch of slides. Select seroreactive proteins were purified and printed on the chip by the manufacturer to serve as positive control spots. Individual serum samples were diluted (1:100) and hybridized to each microarray pad. A biotin-conjugated goat anti-human (IgG) secondary antibody, followed by a fluorescently labelled streptavidin conjugate, were used to probe for IgG bound to antigens on the pads. IgG reactivity was quantified by a microarray scanner (GenePix Pro software) as a unitless relative intensity (Fig. 2). The final hybridization results reported here were performed over 4 days using two different print batches of slides.

### Data analysis

In addition to hybridizations and scanning, data analysis was carried out at the Indian Institute of Technology Bombay (IITB). Normalization was performed as previously described [16]. Median normalized signal intensities (MNSI) were used to generate heat maps, while Log2 FOC values were used for statistical tests. Antigens were considered seroreactive if the average signal intensity for a group of different patients (MSI) was greater than a cut-off, defined as the average plus two standard deviations of the reactivity to all 24 IVTT control spots. Patient antibody breadth was defined as the number of seroreactive antigens per individual, per group, based on the criteria mentioned above. Differentially reactive proteins between two groups were determined using the Statistical Analysis for Microarrays (SAM) module in MetaboAnalyst 4.0. The test was performed assuming equal variance between the groups and an adjusted p value < 0.05 was considered statistically significant. Data was graphically represented using GraphPad Prism (Prism v6.0, GraphPad Software Inc., La Jolla CA).

## Results

### Characteristics of the study population

In order to correlate immune responses with aspects of the local clinical manifestations of malaria, the participant data were grouped into two major classes; *P. falciparum* infected and *P. vivax* infected based on the *Plasmodium* species detected by microscopy. Analysis of these two populations would provide information on immune status of a patient with respect to responses to infection by each of the two human malaria parasite species seen. In addition, both study groups (*P. falciparum* and *P. vivax*) were sub-divided into individual age distributions as shown in Fig. 1a and b. The overall study population had a median age of 25 years (IQR 20–39 years) and was predominantly male (89.5% and 86% male for *P. falciparum* and *P. vivax* groups, respectively, Fig. 1c). This gender bias in seeking hospital care in India has been reported earlier for hospitals in Mumbai and Rourkela [21].

Patients were also sub-divided according to the severity of disease, as immune patterns that correlate with low severity might, in principle, give an indication of disease protecting immunity. According to the clinical information that was documented for each patient, 90/200 patients were hospitalized (in-patients), while 110 participants did not require hospitalization (out-patients) [20]. This number includes patients diagnosed with mixed infections (Fig. 1d). In-patients were further classified into severe (S) and non-severe (NS) groups. Of the 90 in-patients, 64 presented with at least one severe sign/symptom/diseases such as severe anaemia, cerebral malaria, respiratory distress, ARDS, jaundice, convulsions, hypoglycaemia, renal failure and abnormal bleeding. They were classified as patients with severe disease, while in-patients who did not exhibit severe symptoms were non-severe (Fig. 1e). Clinical information on disease severity could not be documented for 107 participants (38 *P. falciparum* and 66 *P. vivax*) who were all out-patients. It is important to reiterate that not all hospitalized individuals exhibited severe symptoms.

According to the World Health Organization guidelines for severe *P. falciparum* malaria, patients with parasite densities > 10% have severe infection, while there is no parasite density threshold for severe *P. vivax* malaria. A majority of the patients in this study presented with moderate to low parasite densities (< 2% or 100,000/µL) except two with parasite densities of 192,153 (*P. falciparum*) and 147,809 parasites/µL (*P. falciparum* + *P. vivax*), estimated by microscopy. Importantly, there was no correlation between parasite density, hospitalization status and severity, thus making study of immune status important (Additional file 1: Table S1).

Patients were further classified based on the time of sample collection. One hundred and twenty-eight patients presented with symptoms during the peak malaria season (May to November), while a separate group of 68 patients were admitted during the dry season (December to April). In order to eliminate sampling bias, the sample details were kept blind to those handling sera hybridizations or microarray slides until after data analysis.

### Identification of seroreactive antigens associated with malaria exposure

**Fig. 3:**
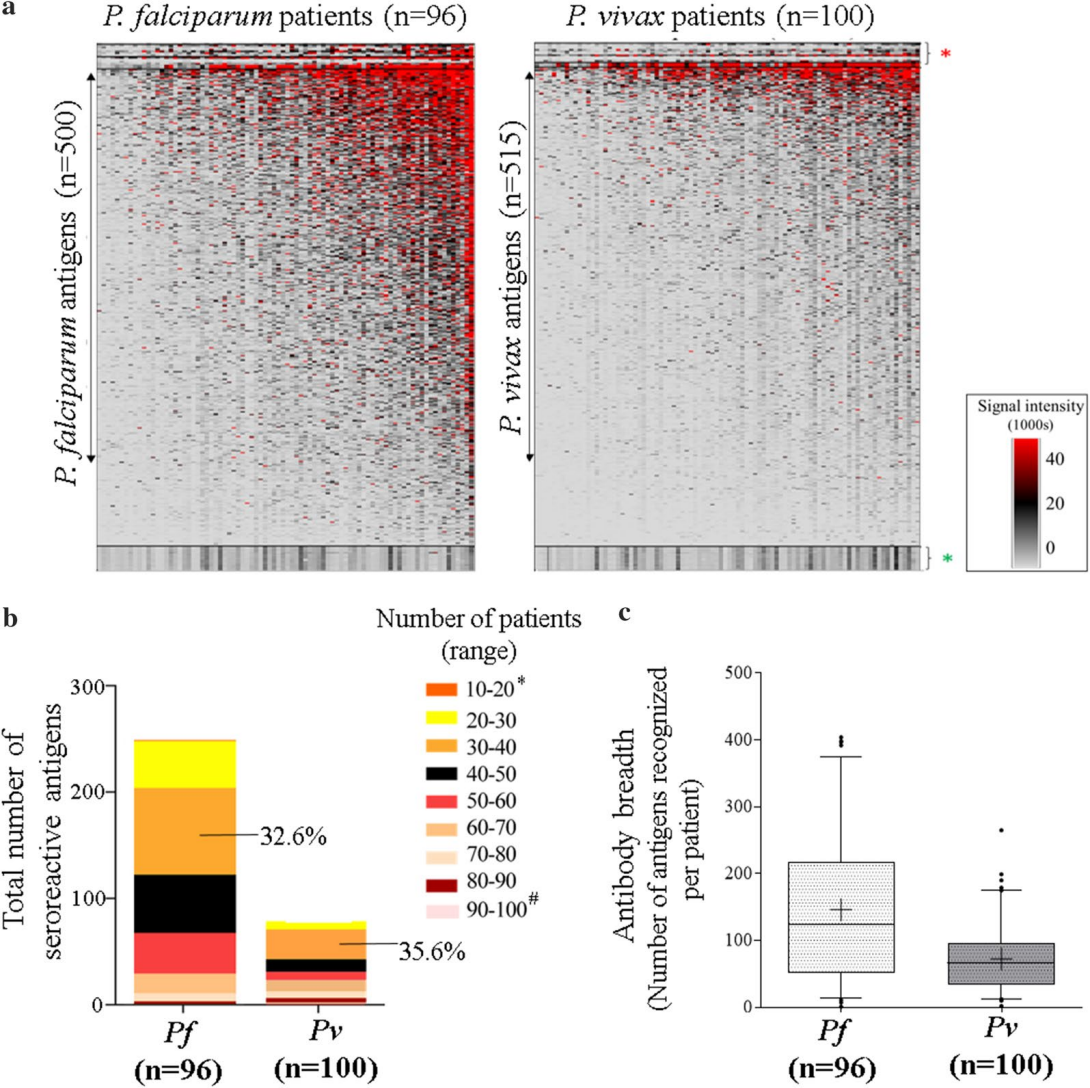
Seroreactivity and breadth of antibody response to *P. falciparum* and *P. vivax* malaria. **a** Heat map showing median normalized signal intensity values (MNSI) of 96 *P. falciparum* and 100 *P. vivax* patients to 500 *P. falciparum* and 515 *P. vivax* polypeptides, respectively. The three-colour gradient indicates high, intermediate and low median normalized signal intensity values. Red indicates highest values above 40,000, while white indicates no reactivity. Patients (columns) are ranked from left to right based on total mean values of antibody binding to all printed polypeptides. Polypeptides are ranked from top to bottom based on their mean values of antibody binding for all serum samples (Samples and polypeptides segregated according to parasite species). *(red)- IgG, anti-human IgG and postive controls, *(green)- 24 No DNA-controls. **b** Bar graph indicates the total number of seroreactive antigens in both study groups (278 *P. falciparum* and 73 *P. vivax*). The colour scale indicates the number of patients (range) seroreactive to a particular percentage of *P. falciparum* and *P. vivax* antigens. (*only in *P. falciparum*, ^#^only in *P. vivax*). **c** Box plots describe antibody breadth to 500 *P. falciparum* and 515 *P. vivax* antigens. The box represents the interquartile range (IQR) with the horizontal line as the median value. The whiskers represent the minimum and maximum values from the data

As detailed in "Methods" section, serum samples from 96 *P. falciparum* and 100 *P. vivax* positive patients were probed on a protein array having 500 *P. falciparum* and 515 *P. vivax* polypeptides (Antigen Discovery, Inc., Irvine, CA). The magnitude and breadth of the Ab response, determined by the number of features recognized, was greater in case of *P. falciparum* than *P. vivax* malaria (Fig. 3a). Two hundred and forty-eight *P. falciparum* and 73 *P. vivax* polypeptides were recognized by *P. falciparum* and *P. vivax* patient groups, respectively (Fig. 3b, Additional file 2: Table S2). A large number of antigens (33%-*P. falciparum* and 36%-*P. vivax*) were found to be seroreactive in 30–40 patients, while only a few antigens (1.2%-*P. falciparum* and 1.4%-*P. vivax)* were seroreactive in more than 80 *P. falciparum* and 90 *P. vivax* patients. Two *P. falciparum* antigens and 8 *P. vivax* antigens showed lowest reactivity among all other seroreactive antigens. These were reactive in 10–20 *P. falciparum* and 20–30 *P. vivax* patients, respectively.

The antibody breadth (calculated by the number of seroreactive antigens in a group of individuals) is shown as a box and whisker plot in Fig. 3c. A few patients showed high levels of Ab binding to as many as 404, 398, 397 and 392 out of 500 *P. falciparum* antigens while one *P. vivax* patient had high reactivity to 265 out of 515 *P. vivax* antigens. Despite the large breadth of antibody responses, these patients were not excluded from the study. The gene IDs of the top antigens showing seroreactivity in more than 60% of the patients in both groups are listed in Table 1A and B.

**Table 1:** Top seroreactive *Plasmodium falciparum* and *Plasmodium vivax* antigens

(A) Top seroreactive *Plasmodium falciparum* antigens					
S. no	Gene ID	ORF fragment	Product description	Molecular weight (kDa)	Sero-reactivity*
1	PF3D7_0422100	–	Transmembrane emp24 domain-containing protein, putative	44.929	86
2	PF3D7_1002100	Exon 2 of 2	EMP1-trafficking protein	69.85	85
3	PF3D7_1007700	Exon 1 segment 2	AP2 domain transcription factor AP2-I	182.664	80
4	PF3D7_0315400	Exon 1 of 1	Conserved Plasmodium protein, unknown function	30.521	77
5	PF3D7_0713900	Exon 1 segment 4	Conserved Plasmodium protein, unknown function	415.228	75
6	PF3D7_0620400	Exon 1 of 1	Merozoite surface protein 10	61.38	75
7	PF3D7_1033200	Exon 1 segment 1	Early transcribed membrane protein 10.2	38.925	73
8	PF3D7_1236100	Exon 2 segment 1	Clustered-asparagine-rich protein	51.533	71
9	PF3D7_1121800	Segment 1	Peptidase M16, putative	238.896	71
10	PF3D7_0530100	Exon 2 of 2	SNARE protein, putative	26.69	71
11	PF3D7_0630600	Exon 2 of 2	Conserved protein, unknown function	110.12	70
12	PF3D7_0800200	Exon 2 segment 1	Erythrocyte membrane protein 1, PfEMP1	330.694	69
13	PF3D7_0935600	Exon 1 of 2	Gametocytogenesis-implicated protein	58.343	68
14	PF3D7_0707700	Exon 1 of 1	E3 ubiquitin-protein ligase, putative	110.778	68
15	PF3D7_0420700	Exon 2 segment 1	Erythrocyte membrane protein 1, PfEMP1	247.609	68
16	PF3D7_0511600	Exon 1 of 1	Apical rhoptry neck protein	24.731	67
17	PF3D7_1468100	Exon 2 segment 1	Conserved Plasmodium protein, unknown function	295.827	65
18	PF3D7_1250600	–	Translation initiation factor eIF-2B subunit beta, putative	78.751	64
19	PF3D7_1436300	Exon 1 Segment 1	Translocon component PTEX150	112.408	63
20	PF3D7_1133400	–	Apical membrane antigen 1	72.041	63
21	PF3D7_0716300	Exon 1 segment 1	Conserved Plasmodium protein, unknown function	60.6	63
22	PF3D7_0903500	Exon 1 segment 1	Conserved Plasmodium protein, unknown function	155.151	61
23	PF3D7_0825700	Exon 1 of 1	Conserved Plasmodium protein, unknown function	36.066	61
24	PF3D7_0818900	–	heat shock protein 70	73.914	61
25	PF3D7_0808200	Exon 1 segment 1	Plasmepsin X	65.113	61
26	PF3D7_1419400	Exon 1 segment 2	Conserved Plasmodium membrane protein, unknown function	262.308	60
27	PF3D7_1346400	Exon 1 segment 3	Conserved Plasmodium protein, unknown function	697.045	60
28	PF3D7_1123400	Exon 1	Translation elongation factor EF-1, subunit alpha, putative	62.88	60
29	PF3D7_1028700	Exon 1 of 1	Merozoite TRAP-like protein	58.084	60

(B) Top seroreactive *Plasmodium vivax* antigens					
S. no	Gene ID	ORF fragment	Product description	Molecular weight (kDa)	Sero-reactivity*
1	PVX_117680	Exon 1 of 2	Hypothetical protein, conserved	128.622	90
2	PVX_081830	Exon 2 of 2	Plasmodium exported protein, unknown function	56.605	89
3	PVX_085590	Exon 1 of 1 segment 5	Hypothetical protein, conserved	515.202	83
4	PVX_116780	Exon 1 of 2	Protein transport protein SFT2, putative	29.194	81
5	PVX_083560	Exon 2 of 2	Plasmodium exported protein, unknown function	34.059	81
6	PVX_113825	Exon 1 of 1 segment 1	Transcription factor with AP2 domain(s), putative	426.497	77
7	PVX_099980	Exon 1 of 1 segment 2	Merozoite surface protein 1	196.119	76
8	PVX_121930	Exon 2 of 2	Plasmodium exported protein, unknown function	32.522	74
9	PVX_118705	Exon 1 of 1	Hypothetical protein, conserved	50.807	74
10	PVX_115450	Exon 1 of 2	Hypothetical protein, conserved	21.409	74
11	PVX_092555	Exon 1 of 6 segment 1	WD domain, G-beta repeat domain containing protein	207.048	70
12	PVX_090230	Exon 1 of 2	Early transcribed membrane protein (ETRAMP)	15.803	69
13	PVX_000930	Exon 1 of 1	Sexual stage antigen s16, putative	15.067	67
14	PVX_097625	Exon 1 of 1	Merozoite surface protein 8, putative	54.74	66
15	PVX_084625	Exon 1 of 1 segment 1	P-type ATPase4, putative	148.895	66
16	PVX_085025	Exon 1 of 1 segment 2	Hypothetical protein, conserved	248.059	64
17	PVX_087670	Exon 1 of 1	Hypothetical protein, conserved	13.356	63
18	PVX_101485	Exon 8 of 8 segment 2	Rhoptry neck protein 3, putative	263.609	61
19	PVX_091935	Exon 2 of 3	Hypothetical protein, conserved	37.095	61
20	PVX_114145	Exon 1 of 1	Merozoite surface protein 10, putative	52.34	60
21	PVX_084305	Exon 1 of 1 segment 1	Zinc finger protein, putative	191.964	60

* Indicates number of patients; *Pf* (n=96), *Pv* (n=100). A cut-off of 60% was applied to select the top seroreactive antigens

An analysis of the features/functions of the 248 *P. falciparum* seroreactive antigens indicated that a majority of the seroreactive *P. falciparum* antigens were conserved *Plasmodium* proteins (17.9%) (Additional file 3: Fig S1). The next major category was protein binding (16%), followed by nucleic acid binding (6.6%) and host cell surface binding proteins (5.4%), plus others such as *Plasmodium* exported proteins, MSPs and HSPs. Several enzymes (4.6%), transcription and translation factors (3.5%), proteins involved in ATP-binding (3.9%), ion-binding (3.5%) and proteins involved in ubiquitination (1.5%) also elicited high IgG responses despite their intracellular locations in the nucleus and the cytosol. Antigens with features and functions similar to *P. falciparum* antigens were also seroreactive in *P. vivax* patients, along with hypothetical proteins that constituted the major group (33.3%).

**Fig. 4:**
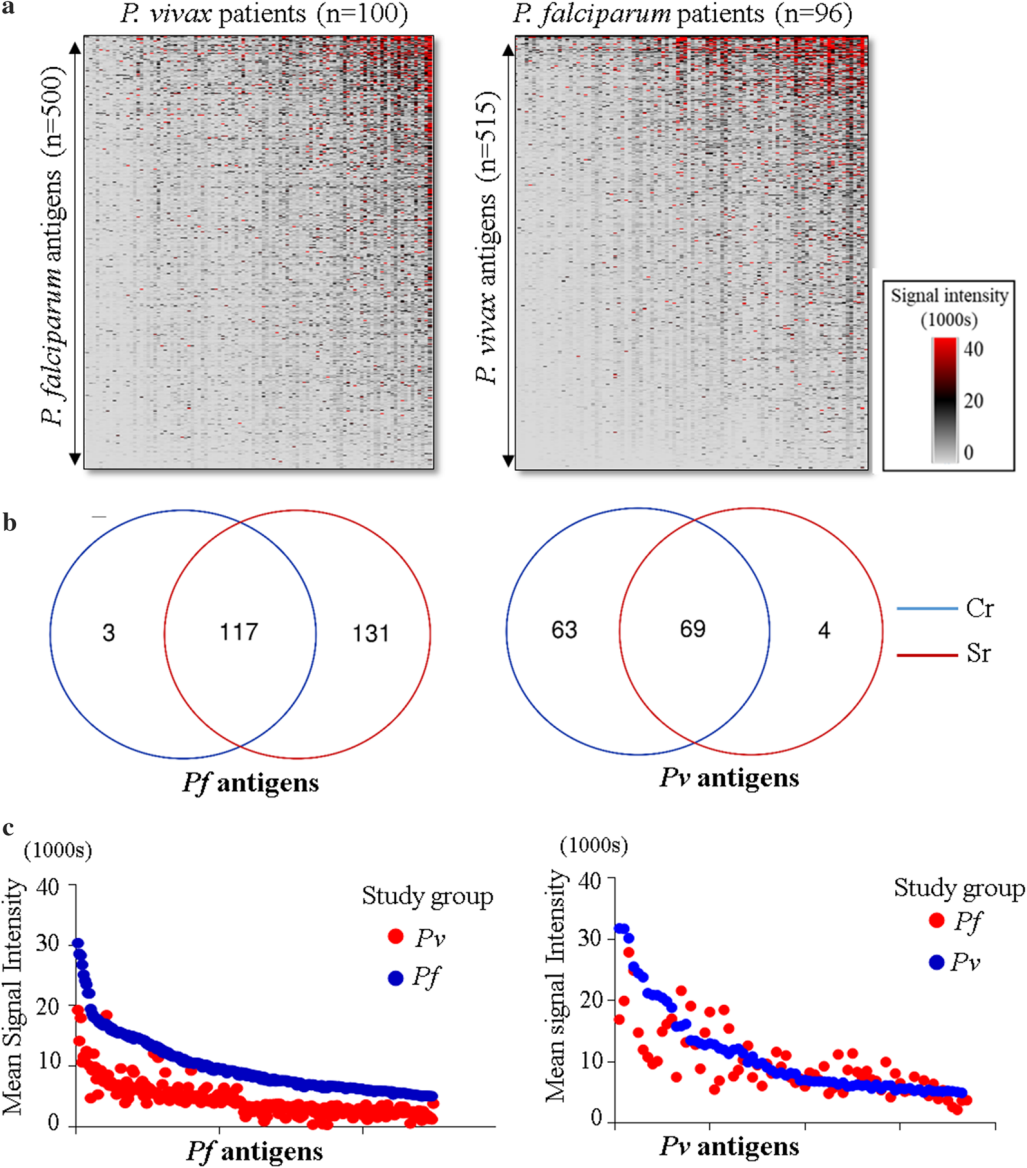
Crossreactivity to *P. falciparum* and *P. vivax* antigens. **a** Heat map showing MNSI of 96 *P. falciparum* and 100 *P. vivax* patients to 515 *P. vivax* and 500 *P. falciparum* polypeptides, respectively. **b** Venn diagram indicates number of *P. falciparum* and *P. vivax* polypeptides that are (i) exclusively crossreactive (Cr) (ii) both crossreactive and seroreactive (Sr) and (iii) exclusively seroreactive. ADI spot IDs (gal file) were used instead of PlasmoDB_IDs in order to distinguish between the different ORF fragments (polypeptides). **c** Scatter plots of average SI of median normalized values (MSI) for all *P. falciparum* and *P. vivax* antigens in both groups

It can be observed from heat maps in Fig. 4a that there was also a high level of cross-reactivity between *P. falciparum* and *P. vivax* antigens but all of the patterns are not easy to understand. One hundred and seventeen seroreactive *P. falciparum* antigens (Sr) from *P. falciparum* patients were also recognized by *P. vivax* patients (Fig. 4b). These antigens are here forth called cross-reactive antigens (Sr&Cr). Strangely in case of *P. vivax* antigens, the number recognized by *P. falciparum* patients was actually more than the antigens recognized by *P. vivax* patients themselves (Fig. 4b). This indicates that overall immune activity of *P. falciparum* patients was higher than the *P. vivax* study group. The cross reactivity with *P. vivax* antigens was especially high in the few *P. falciparum* patients who also showed high reactivity to a large number of *P. falciparum* antigens. While 131 out of 248 *P. falciparum* antigens were exclusively reactive in the *P. falciparum* patient group, a similar observation could be made only for 4 out of 73 *P. vivax* antigens (Fig. 4b). Gene annotations for the data represented in the Venn diagrams are mentioned in Additional file 4: Table S3. A scatter plot of MNSI levels for all *P. falciparum* and *P. vivax* antigens in both groups is shown in Fig. 4c to depict the level of crossreactivity observed in the study. Deeper analysis yielded an even more unexpected finding. All antigens that were reactive in both *P. falciparum* patients and *P. vivax* patients, shared orthologs with other *Plasmodium* species (PlasmoDB), including non-human infecting parasites. However, 29 *P. falciparum* antigens did not have orthologs in *P. vivax*-Sal1 strain. On the contrary, 98 *P. falciparum* antigens which were reactive only in *P. falciparum* group (not crossreactive in *P. vivax* patients) had orthologs in *P. vivax* (Additional file 5: Table S4, last tab). Likewise, few crossreactive *P. vivax* antigens did not share orthologs with *P. falciparum*-3D7 strain. Data for orthology and synteny are presented in Additional file 5: Table S4.

### Age dependent alterations in antibody levels to *Plasmodium* species

In order to distinguish between recent and cumulative exposure, patients were further segregated based on age into four sub-groups as mentioned previously (1–10 years-A, 11–22 years-B, 23–39 years-C and 40–65 years-D). The sample size differed in each age group as follows: The number of falciparum patients were 5, 36, 36 and 10, while vivax patients were 9, 30, 34 and 27 patients in age groups A, B, C and D, respectively. Clearly, the study involved some groups with very small numbers, especially children aged 1 to 5 years. Hence, a comparison of seroreactivity between groups was not performed. Moreover, there was no correlation between the number of seroreactive proteins and patient age, when scatter plots depicted antibody breadth for individual patients (Additional file 6: Fig S2). Instead of a statistical analysis between age groups, individual groups were analysed separately to identify top seroreactive antigens (Additional file 7: Table S5). The main aim was to determine if any of the antigens were unique to a particular age group. Based on early work, much from Africa, it is clearly known that children aged 0–5 years are immunologically different from other groups. In India, this is true for some but not all reactive antigens. As anticipated, certain antigens such as HSP40, ACS6, GCVH, among others, were seroreactive only in children. Likewise, several conserved *Plasmodium* proteins, family of tetratricopeptide repeat proteins, SET1, PK4, GPI1, ApiAP2, CSP, SERA7, PUF1, HSP90, HSP70, *Pf*EMP1 family, and several enzymes like helicases, peptidases and methyltransferases were seroreactive only in older adults (> 40 years). These antigens could represent markers of cumulative exposure. Alternatively, certain 88 *P. falciparum* antigens were found to be seroreactive in all age-groups; they included transmembrane emp24 domain-containing protein, ApiAP2, several conserved *Plasmodium* proteins, MSPs, PF70, members of ETRAMP family, liver stage antigens, rhoptry neck proteins, SYN6, PIESP2, members of the PfEMP1 family, enzymes and proteins involved in translation and transcription, rifin and a few cytoskeleton proteins like dynein heavy chain. In case of *P. vivax,* 41 antigens were seroreactive in all age groups, most of which were hypothetical proteins and merozoite surface proteins, while other hypothetical proteins and other known proteins such as MSPs and HSPs were exclusively seroreactive in older adults (Additional file 7: Table S5).

### Serological markers associated with severe and non-severe malaria

**Fig. 5:**
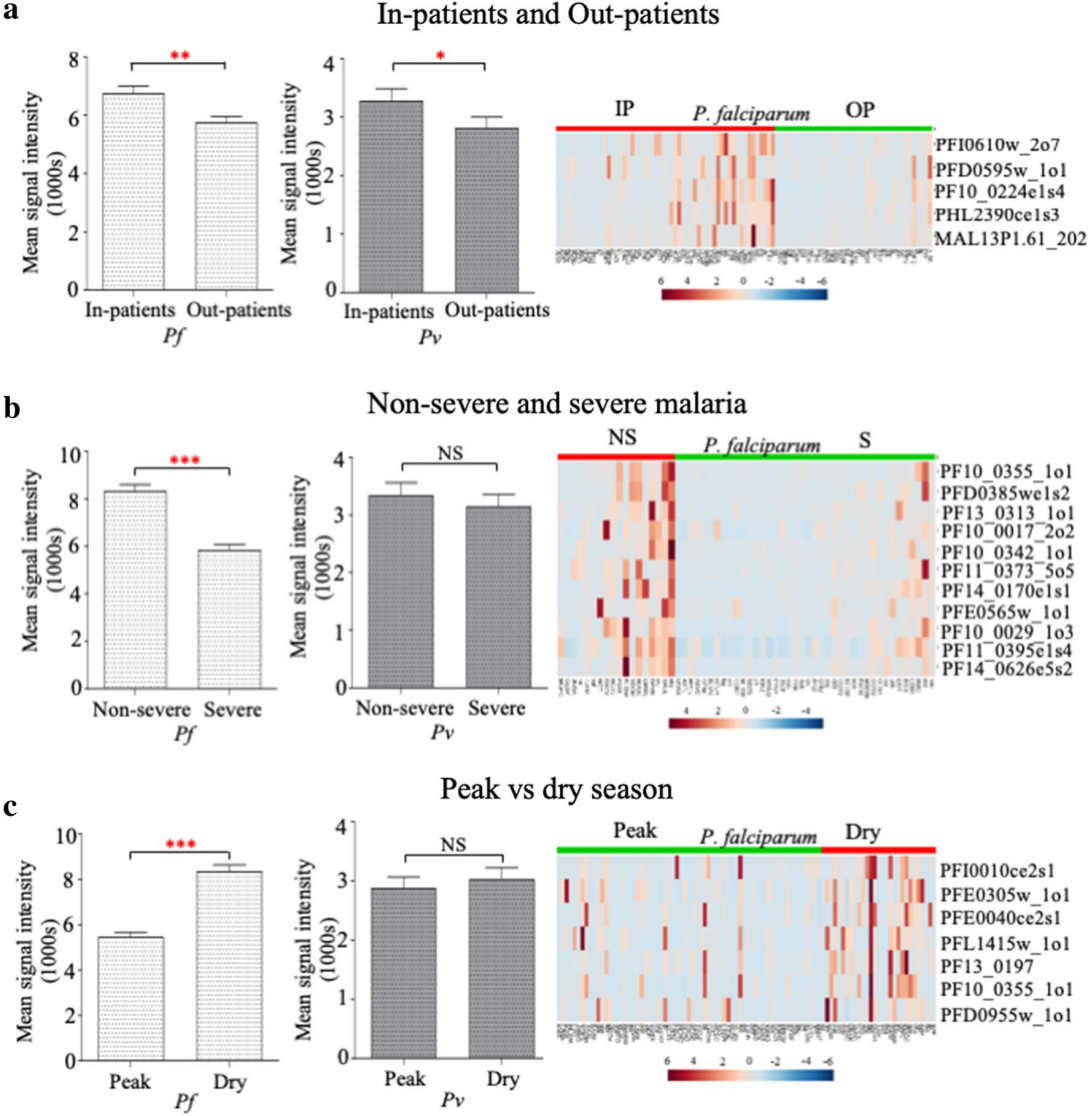
Differential antibody responses among patients classified on the basis of disease: **a** hospitalization status, **b** severity symptoms and **c** time of sample collection. The bar graphs on the left indicate average SI of median normalized values (MSI) for each study group with 95% CI. **p < 0.001, ***p < 0.0001, *0.001 < *p *< 0.05, *NS* not significant (Mann–Whitney non-parametric statistical test). Heat maps on the right represent MNSI values (autoscaled) of individual patients (*P. falciparum* only) to specific *P. falciparum* antigens which showed differential antibody response between **a** in-patients and out-patients, **b** non-severe and severe patients, and **c** patients diagnosed with *P. falciparum* malaria during peak malaria season and dry season. Significance Analysis of Microarrays (SAM) using Log2 FOC values was performed to identify statistically significant proteins with *p * < 0.001. Only antigens that were seroreactive in at least one group are represented in the heat map

Malaria positive falciparum and vivax patients were segregated into in-patients and out-patients as mentioned previously. The in-patients showed a significantly higher antibody response compared to out-patients in both *P. falciparum* (*p* = 0.002) and *P. vivax* (*p* = 0.01) study groups, respectively. SAM analysis using Log2 FOC values revealed a list of five candidate *P. falciparum* antigens with differential seroreactivity among the two *P. falciparum* sub-groups sensitive to hospitalization (p < 0.001, Fig. 5a, Table 2). Three antigens were conserved *Plasmodium* proteins and two others were dynein heavy chain and *Plasmodium* exported protein (GEXP07). In case of *P. vivax,* a hypothetical protein and ubiquitin-like protein (p < 0.001, Table 2) showed differential seroreactivity with hospitalized patients.

**Table 2:** Antigens with differential seroreactivity in patients classified based on hospitalization, severity status and time of sample collection

S. no	PlasmoDB_ID	ORF_fragment	Product description	MSI		*p* value^a,b^
				In-patients	Out-patients	
1	PF3D7_0912500	Exon 2 of 7	Conserved Plasmodium protein, unknown function	8142.5	3291.5	0.0010
2	PF3D7_0412000	Exon 1 of 1	Conserved Plasmodium protein, unknown function	7824.2	3517.4	0.0012
3	PF3D7_1023100	Exon 1 segment 4	Dynein heavy chain, putative	7769.7	3347.2	0.0024
4	PF3D7_1249800	Exon 1 segment 3	Conserved Plasmodium protein, unknown function	6368.7	2157.0	0.0019
5	PF3D7_1301700	Exon 2 of 2	Plasmodium exported protein (hyp8), unknown function (GEXP07)	5154.5	1742.9	0.0034
1	PVX_083040	Exon 2 of 2 segment 1	Hypothetical protein, conserved	9916.3	3654.6	0.0000
2	PVX_099520	Exon 3 of 8	Ubiquitin-like protein, putative	6774.6	2716.2	0.0001

S. no	PlasmoDB_ID	ORF_fragment	Product description	MSI		*p* value^a,b^
				Non-severe	Severe	
1	PF3D7_1036300	Exon 1 of 1	Merozoite surface protein (MSP3.8)	11,334.2	3432.4	0.0168
2	PF3D7_0407800	Exon 1 segment 2	Conserved Plasmodium protein, unknown function	13,110.6	3767.8	0.0176
3	PF3D7_1358500	Exon 1 of 1	Zinc finger protein, putative	9686.6	2319.6	0.0025
4	PF3D7_1001300	Exon 2 of 2	Plasmodium exported protein (PHISTa), unknown function	9143.6	3848.5	0.0174
5	PF3D7_1035100	Exon 1 of 1	Probable protein, unknown function	8142.7	1980.5	0.0197
6	PF3D7_1136200	Exon 5 of 5	Conserved Plasmodium protein, unknown function	9915.7	3659.9	0.0111
7	PF3D7_1417200	Exon 1 segment 1	NOT family protein, putative	9811.8	3083.4	0.0133
8	PF3D7_0511400	Exon 1 of 1	Conserved Plasmodium protein, unknown function	10,704.2	2997.8	0.0212
9	PF3D7_1002500	Exon 1 of 3	Conserved Plasmodium protein, unknown function	8133.8	2635.9	0.0045
10	PF3D7_1138400	Exon 1 segment 4	Guanylyl cyclase (GCalpha)	5815.1	2521.4	0.0152
11	PF3D7_1465800	Exon 5 segment 2	Dynein beta chain, putative	5733.4	1942.9	0.0136

S. no	PlasmoDB_ID	ORF_fragment	Product description	MSI		*p* value^a,b^
				Peak season	Dry season	
1	PF3D7_0900200	Exon 2 segment 1	Rifin (RIF)	4770.0	11,917.5	0.0053
2	PF3D7_0506200	Exon 1 of 1	Transcription initiation factor TFiid, TATA binding protein (TBP)	4883.8	10,961.1	0.0027
3	PF3D7_0500800	Exon 2 segment 1	Mature parasite infected erythrocyte surface antigen, erythrocyte membrane protein 2 (MESA)	3575.5	12,533.2	0.0000
4	PF3D7_1229300	Exon 1 of 1	conserved Plasmodium protein, unknown function	4564.7	10,429.6	0.0049
5	PF3D7_1335100	–	Merozoite surface protein 7 (MSP7)	3391.6	12,143.4	0.0006
6	PF3D7_1036300	Exon 1 of 1	Merozoite surface protein (MSP3.8)	3930.7	10,821.2	0.0011
7	PF3D7_0419700	Exon 1 of 1	Apical merozoite protein (Pf34)	4112.4	9532.6	0.0079

^a^*p* value using SAM analysis, ^b^ Data shown for *Pf* antigens only for all comparisons, except in case of hospitalization status

When in-patients were further classified into two groups, severe (S) and non-severe (NS), non-severe falciparum patients displayed significantly higher Ab levels compared to severe falciparum patients (*p* < 0.0001). It is possible that some of these antigens could offer protection from severe disease, although many more diverse clinical events will have to be characterized to fully test this hypothesis. Several conserved *Plasmodium* proteins, MSP 3.8, PHIST a, NOT family protein and a few others were found to be seroreactive only in non-severe patients (*p* < 0.001, SAM, Fig. 5b, and Table 2).

### Serological markers associated with seasonal malaria

Goa is characterized by seasonal malaria transmission, yet many clinical cases are also reported during the dry season. To study the dynamics of IgG Ab responses accompanying changes in intensity of malaria incidence and transmission, patients were segregated based on time of the year during sample collection. A marked increase in seroreactivity was observed during the dry season (post-malaria season) in *P. falciparum* patients (*p* < 0.001, SAM). Most of these antigens were seroreactive in both seasons, but a few *P. falciparum* antigens (e.g. TBP, *Pf*34, MESA and rifin, along with MSPs and conserved *Plasmodium* proteins), had higher Ab responses in select patients during the dry season, (Fig. 5c and Table 2). The reasons underlying this observation are not known. In the case of *P. vivax* infections, only a few patients (66%) were seroreactive in both wet and dry seasons and 31% were seroreactive only during peak wet season.

## Discussion

Naturally acquired immunity (NIA) depends on previous infection and builds with every pathogen encounter [22]. It is generally proportional to the duration and degree of exposure to parasites, wanes rapidly in the absence of an active infection [23, 24] and is specific to the infecting *Plasmodium* species. To an extent, it is also specific to different life stages of the parasite [22]. To disease control experts, NAI, especially in children can be useful for estimating areas with on-going transmission and varying disease burden [25]. In principle, it may be used to determine whether populations are at a high risk of severe disease.

India contributes significantly to the overall global malaria burden. It is important for the study of malaria immunity due to (a) the diverse eco-epidemiological profiles across the country, (b) co-existence of multiple *Plasmodium* species and vectors, (c) changing climatic patterns that may have an impact on malaria transmission and (d) emergence of anti-malarial drug resistance [26]. India has an extensive integrated public health surveillance system to identify disease burden, as well as morbidity and mortality in a community [27]. As a part of an early founding inter-ICEMR collaboration, a study described the serological profiles of 236 malaria positive patients from three different sites in India. Indian samples showed seroreactivity to 265 *P. vivax* and 373 *P. falciparum* antigens. A significant difference in the levels of seroreactivity and breadth of antibody response was observed across the three study sites [19]. Additionally, a linear correlation was observed between the breadth of Ab response and malaria prevalence with respect to *P. falciparum,* but not *P. vivax*. These findings suggest that indicators of exposure may vary across diverse endemic settings and there may be a need for establishing pan-specific general antigens that may be of use for surveillance campaigns in India [19]. Of particular interest are urban and semi-urban areas that have local malaria transmission but also large movement of workers from rural and poorer states of India [20].

The current study focuses on patients from Goa, a small prosperous coastal state in western India where the epidemiology of malaria includes the presence of migrant workers and transient communities. Goa is classified as a non-endemic region with low transmission intensities where parasitaemia are usually low (< 1%). Using the shared ICEMR protein array platform, the present study describes the profiles of NAI in malaria-positive patients residing in Goa. The primary goal was to explore species-specific and pan-species serological correlates of exposure. Further, differential seroreactivity was identified in severe versus non-severe malaria patients to help identify antigens likely to offer protection from severe manifestations. This cross-sectional serological study was performed using a small subset of the total number of malaria-positive individuals referred to the MESA-ICEMR study team [20]. Of over 1000 confirmed malaria cases, a majority (88.2%) were born outside of Goa (primarily Uttar Pradesh and Bihar) and 51% were construction workers. A larger proportion of the patients were diagnosed with *P. vivax* (77%), while 21% had *P. falciparum* malaria. Of these 1000 confirmed malaria cases at GMC, 96 falciparum and 100 vivax patient sera were used in the present serology study.

Consistent with previous ICEMR protein array data from around the world, *P. falciparum* patients showed a remarkably stronger immune response compared to *P. vivax*. The reasons for this could be many, the major one being the significantly different biology of the two parasites [16]. Secondly, the protein arrays were designed against the genome of a single strain for each species (*P. falciparum* 3D7, *P. vivax* Sal1), allowing the introduction of several important biases. Genetically, *P. vivax* is extremely diverse and the protein array is made based on a South American Strain. This may explain the lower general reactivity seen in Goa. Overall, 248 *P. falciparum* and 73 *P. vivax* seroreactive proteins were identified. Interestingly, most of the top seroreactive antigens of *P. falciparum* (Table 1A) were previously identified in global studies using *P. falciparum* protein array platforms [11, 15, 28, 29]. Many were also found to be immunogenic in more rural endemic regions of India [19]. Similarly, *P. vivax* antigens have also been seen in previous global microarray studies from other parts of the world. According to a previous study, a few *Plasmodium* exported proteins (PVX_083560, and PVX_121930), a hypothetical protein (PVX_118705) and MSP10 could discriminate between naïve and semi-immune individuals [12]. The immuno-proteome of *P. vivax* recently published using a protein array with 1936 genes encoding *P. vivax* proteins identified 151 highly seroreactive *P. vivax* antigens, a few of which (PVX_115450, PVX_090230, PVX_085025 and PVX_087670) were also recognized in our study [14]. In conclusion, for global prioritization of antigens, select proteins transcend national and continental variations in malaria host-parasite biology.

Importantly, the malaria patients in Goa showed differential levels of IgG reactivity to different polypeptides from the same protein, in both *P. falciparum* and *P. vivax*. Individual proteins, or even different peptides in a protein, can elicit varying levels of IgG reactivity. Protein-specific features such as subcellular location, protein abundance, degree of polymorphism and presence of human orthologs may influence the magnitude of antibody responses during natural malaria infections [17]. Overall, once an antigen receives high priority based on epidemiological investigations, a deeper hunt for the best antigenic peptide from that protein may be fruitful for signal intensity and breadth of responses in target patient populations.

A majority of the 248 *P. falciparum* and 73 *P. vivax* seroreactive antigens identified were understandably either proteins normally exported to the erythrocyte cell surface during the parasite life cycle or present on the merozoite surface. Yet, some nuclear, cytoskeletal and cytoplasmic proteins also triggered high IgG binding in the majority of the infected population. A 1990s study from India, provided early evidence for immune reaction against intracellular proteins [30]. A differential immuno-screening of an erythrocyte-stage cDNA expression library revealed novel protein targets that were exclusively recognized by immune sera and not acute patient sera. Surprisingly, one was a conserved ribosomal protein P0 and another was an endonuclease, alongside several conserved hypothetical proteins. Antibodies against four of these proteins also inhibited *P. falciparum* growth in culture and correlated with protection in mice [30]. Most important invasion proteins, and also those strongly implicated in correlates of protection, are highly polymorphic. This may reduce seroreactivity and introduces a bias in protein screens. The latter may favour highly conserved genes which are often intracellular but not necessarily relevant for protection.

The basis of the major cross-reactivity observed in the study could be the large number of orthologous *P. falciparum* and *P. vivax* proteins printed on the chip, but it is not that simple. Of 123 *P. falciparum* antigens exclusively seroreactive in the *P. falciparum* study group, 93 were found to share orthologs in *P. vivax* (Additional file 5: Table S4). Reciprocally, there were also non-orthologous antigens which did not show any species specificity. These observations highlight the need to better understand antigenic cross-reactivity. It should be noted that differentiation between *P. vivax* and *P. falciparum* co-infections was based on two independent microscopy determinations. It is possible that cross-reactivity due to sub-microscopic infection could affect antibody levels in areas such as Goa, where both *P. falciparum* and *P. vivax* co-exist. Finally, residual seroreactivity from past exposures to either *P. falciparum* or *P. vivax* could also be responsible for unaccounted cross-reactivity.

In areas of high transmission, parasitaemia and risk of morbidity and mortality have an inverse relationship with age: Disease versus parasitaemia can be distinct, and dependant on age. Children often exhibit anti-disease immunity, which confers protection from the risk and extent of morbidity associated with a given parasite density [22]. In contrast, adults exhibit anti-parasite immunity which protects against high parasite density and the risk of severe disease. Compared to the above patterns in high-transmission areas, immunological patterns can be different in areas with low transmission. In our study, a few antigens showed remarkably higher signal intensities in older adults. Higher reactivity in adults can arise from legacy antibody profile derived from decades of previous intense exposure, which is durable or can arise with age. To further complicate matters, our study is dominated by a migrant population, so this finding could be the product of both historic exposure from a different epidemiological setting and recent exposure boosting select parts of the NAI. It is noteworthy that the common responses transcend epidemiological settings and are age dependent, and not the result of several years of heavy local exposure in Goa [22].

To explore the possibility of identifying markers of disease protective immunity, participants were segregated as in-patients and out-patients. The overall antibody response was higher in in-patients compared to out-patients, and this was presumably a response to the most recent infection (Fig. 5a). In-patients were further classified as severe and non-severe based on the signs/symptoms of severity. Importantly, a few *P. falciparum* antigens were recognized in all sera from non-severe hospitalized patients, and were largely non-reactive in most of the severe hospitalized sub-group. These antigens could serve as correlates of protection against severe manifestations or may even confer protection against disease. Although further assessment is required to fully understand immune responses that can protect against disease, this study provides the first evidence of differential seroreactivity to *P. falciparum* antigens in severe and non-severe patient cohorts using protein microarrays. This trend was not observed in case of *P. vivax* malaria, possibly due to the overall low antibody response to *P. vivax*. Deeper longitudinal cohort studies will be necessary to expand the body of evidence for such potential protective immunity.

With the success of malaria control programmes, transmission is likely to decline in the next few years. Low transmission areas require increasingly sensitive tools to check for malaria exposure. Antigens which are seroreactive across varying epidemiological and transmission settings, and exhibit species-specificity may represent the best markers of exposure. Protein arrays provide a suitable platform for the identification of a manageable subset of antigens eliciting the strongest response. Antibodies against these antigens may be measured using basic immunoassays, such as enzyme-linked immunosorbent assays (ELISA). The biggest benefit is the ease with which antibodies can be stored using dried blood spots, making sample collection and storage relatively simple [31].

To build on present advances, future follow-up studies may improve in four areas: first, more effort is required to enroll children and adolescent patients. Second, longitudinal serology studies will track exposure to malaria, protective immunity, and waning of such immunity after an infection is resolved. Third, more detailed information will be sought on the origin of migrant workers and prior history of infection in their place of origin. Finally, the array platform itself will evolve to test sera against some shorter exon fragments on the array, some full-length proteins, all accepted blood-stage antigens, and antigens from non-erythrocytic stages of malaria parasites to assess their contribution to local transmission.

## Conclusions

Malaria exposure is largely underestimated in several countries due to limitations in microscopy-based diagnosis. The present study provides insights into antibody-based immune responses against parasite antigens in low transmission settings in India, using data collected over 4 years during both peak and dry malaria seasons. The work complements global efforts by ICEMR programs to compare variations in malaria across the globe, including possible variations in immunity to malaria. The overall immunity patterns in Southwestern India mirror what has been seen in other parts of India and the world. The contrasting immunity in individuals with varying disease presentations at a tertiary care hospital offer powerful new avenues to achieve protective immunity against severe disease.

## Additional files

**Additional file 1.** Patient data for parasite density, hospitalization status and severity.

**Additional file 2.** Seroreactive *P. falciparum* and *P. vivax* antigens.

**Additional file 3.** Functional classification of seroreactive *P. falciparum* and *P. vivax* antigens.

**Additional file 4.** Details of cross-reactive *P. falciparum* and *P. vivax* antigens.

**Additional file 5.** Data for orthology and synteny.

**Additional file 6.** Age-dependent seroreactivity of *P. falciparum* and *P. vivax* antigens.

**Additional file 7.** Scatter plots for antibody breadth in patients.
