# Supplementary figures and images for: Hospital-derived antibody profiles of malaria patients in Southwest India
Source: Malar J. 2019 Apr 17;18:138. doi: 10.1186/s12936-019-2771-5 (PMC6472095; doi:10.1186/s12936-019-2771-5)

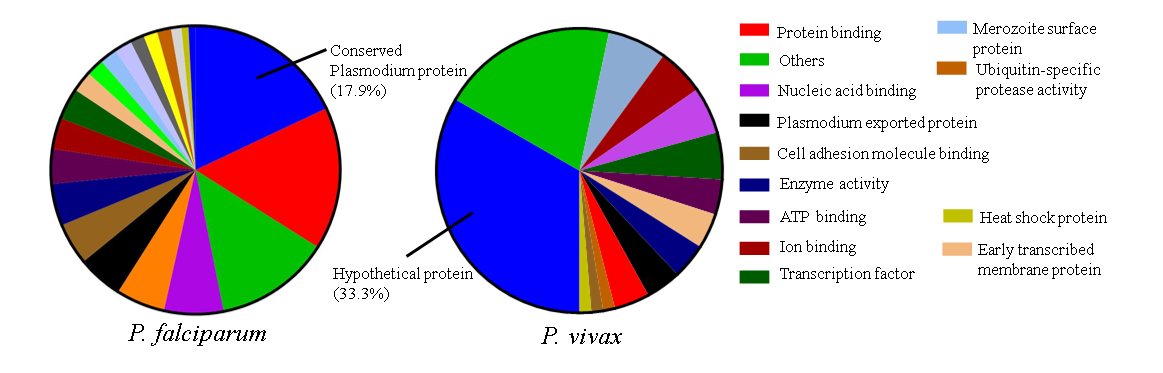

Supplement: Supplementary file 3 — Additional file 3. Functional classification of seroreactive P. falciparum and P. vivax antigens. [file 12936_2019_2771_MOESM3_ESM.png]

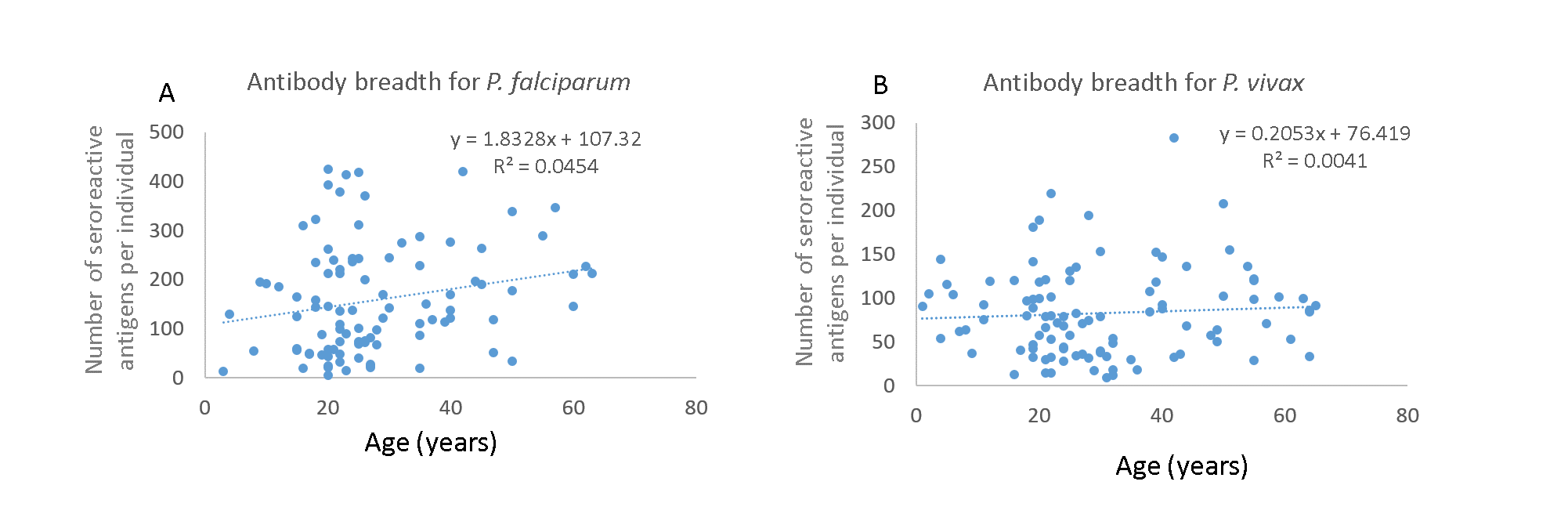

Supplement: Supplementary file 6 — Additional file 6. Age-dependent seroreactivity of P. falciparum and P. vivax antigens. [file 12936_2019_2771_MOESM6_ESM.png]
